# Biological age as estimated by baseline circulating metabolites is associated with incident diabetes and mortality

**DOI:** 10.1016/j.jnha.2023.100032

**Published:** 2024-01-02

**Authors:** La-or Chailurkit, Nisakron Thongmung, Prin Vathesatogkit, Piyamitr Sritara, Boonsong Ongphiphadhanakul

**Affiliations:** a Department of Medicine, Faculty of Medicine, Ramathibodi Hospital, Mahidol University, Bangkok, Thailand; b Research Center, Academic Affairs and Innovations, Faculty of Medicine, Ramathibodi Hospital, Mahidol University, Bangkok, Thailand

**Keywords:** Metabolomics, Diabetes, Mortality, Biological age, Chronological age

## Abstract

**Objectives:**

It is unclear how metabolomic assessment of biological aging performs in non-White populations and whether such an approach can predict future mortality. We aimed to evaluate the application of serum metabolomics combined with machine learning methodologies to predict incident diabetes and mortality in a Thai population.

**Design, setting and participants:**

We analyzed serum samples and mortality data over 11 years from among 454 participants with no previous history of diabetes and with a fasting plasma glucose ≥85th percentile (5.4 mmol/L) but <7 mmol/L.

**Measurements:**

Untargeted serum metabolomics were assessed using liquid chromatography/mass spectrometry. A deep artificial neural network was used to predict biological age based on serum metabolite profiles and chronological age.

**Results:**

The mean age of participants was 40.5 ± 6.4 years, and 70.8% were men. We found a significant positive correlation between metabolomic age and chronological age (r = 0.71, P < 0.001). After 5 years, 61 of 404 participants with available glycated hemoglobin status (15.1%) progressed to diabetes. Chronological age was associated with incident diabetes but was not significant (P = 0.08), after adjusting for BMI and sex. Metabolomic age was significantly related to incident diabetes after controlling for BMI and sex (P < 0.05). Over the 11-year follow-up, 10 participants died owing to non-accidental causes. When metabolomic age and chronological age were included together in the model, metabolomic age (but not chronological age) was associated with mortality, independent of age, sex, and BMI. Among all identifiable metabolites, beta-D-mannosylphosphodecaprenyl and phosphatidylserines were the five leading metabolites associated with mortality.

**Conclusion:**

We concluded that serum metabolomic profile was associated with incident diabetes as well as mortality over our 11-year study period, which may render it potentially useful in assessing biological aging in humans.

## 1 Introduction

Aging is a generalized process resulting in a decline in physiological function and eventually leading to increased morbidity and mortality [1]. In general, chronological age is used as a proxy for the aging process, and has been shown to be rigorously associated with life expectancy as well as adverse conditions related to aging [2]. However, at the level of an individual, there are significant variations in physiological functions among subjects with the same chronological age. The idea of ‘biological age’ emerged in response to this, aiming to quantify the actual physiological and biological state of an individual, as opposed to the mere passage of time since birth. Biological age is defined through various evaluative methodologies, including biomarkers of aging, epigenetic clocks, and functional assessments, thus offering a more nuanced and personalized understanding of an individual’s aging trajectory [3]. Moreover, the discordance between chronological and biological age has been put forward as an additional tool for assessing an individual’s susceptibility to the aging process. This disparity, often influenced by multifactorial components such as genetic factors, lifestyle behaviors, and exposure to environmental stressors, could serve as an invaluable prognostic marker to predict health trajectories and customize intervention strategies [4,5].

Numerous markers or hallmarks have been identified that can provide insights into biological aging, such as telomere length, DNA methylation patterns, and cellular senescence [6]. These indicators reflect the physiological status of an individual beyond their chronological age, thus offering a more comprehensive assessment of health status and disease risk. Recently, advances in the field of metabolomics have paved the way for the use of circulating metabolomes, the complete complement of small compound metabolites (<1500 Da) found within a biological sample, as predictors of biological processes as well as tools to further understanding of involved mechanisms including biological age [7]. In a cohort study conducted in the United Kingdom (UK), metabolomic markers were demonstrated to have predictive performance for certain diseases that is on par with aging clocks derived from DNA methylation patterns [8]. However, the generalizability of these findings remains an open question. Current literature is insufficient to clarify how such metabolomic assessment of biological aging performs across diverse populations, especially among non-White groups. This knowledge gap is particularly significant as genetic, environmental, and lifestyle factors can vary widely among different populations, which could potentially influence the efficacy of metabolomic markers. Furthermore, the potential of these metabolomic markers in predicting long-term outcomes like mortality is not well understood.

To address these issues, the present study intends to evaluate the application of serum metabolomics combined with machine learning methodologies to predict incident diabetes and mortality over an 11-year follow-up period in a Thai population. By expanding our understanding to different demographic groups, this study seeks to enrich the evidence base around the practical use of metabolomic markers in understanding biological aging and disease susceptibility.

## 2 Methods

### 2.1 Participants

Participants were recruited from among participants in the Electricity Generating Authority of Thailand (EGAT) study in 2009, cohort 3 (EGAT 3). Details of the study cohort have been published previously [9]. Briefly, participants in the cohort were employees of EGAT who volunteered to participate in a health survey. All participants completed a medical evaluation and had routine laboratory tests, including urinalysis. Demographic data including age, sex, marital status, education level, body mass index (BMI), blood pressure and smoking status were collected. Blood was drawn after a 12-h fast. There were three EGAT cohorts. In 1985, 3499 workers at EGAT (half of the total employees) were randomly enrolled as the EGAT 1 cohort. In 1998, 2999 employees were randomly enrolled as the EGAT 2 cohort. In 2009, 2584 participants were recruited into the EGAT 3 cohort, and the EGAT 3 cohort was resurveyed in 2020. In each survey, the same individuals were contacted by telephone and invitation letter to attend a follow-up examination. Information about the causes of death was sought for those known to have died during the interim period. At each follow-up visit, participants underwent similar medical evaluations and had routine laboratory investigations, as in the baseline visit.

For this study, a total of 454 participants from the EGAT 3 cohort were randomly selected using computer-generated random numbers. Participants included in the study were individuals aged between at least 25 years at baseline who presented with fasting plasma glucose (FPG) levels ranging between 5.4 mmol/L and 7.0 mmol/L at the time of enrollment, displayed a willingness to participate and provide informed consent. The study excluded individuals who had been previously diagnosed with type 1 or type 2 diabetes and individuals with acute illnesses at baseline. From among participants who did not have a previous history of diabetes and who had a fasting plasma glucose (FPG) ≥85th percentile (5.4 mmol/L) but <7 mmol/L. By choosing a FPG threshold of ≥85th percentiles which results in a slightly lower limit of 5.4 mmol/L, we aimed to capture a wider population that is on the cusp of entering the IFG range (5.6 mmol/L) which could result in a higher sensitivity for capturing subjects with higher risk for developing diabetes. Body mass index (BMI) was calculated using body mass (kg)/height (m)^2^. Serum samples were obtained and kept at −80 °C until analysis.

## 3 Untargeted metabolomic assessment

1) **Sample preparation**: serum was separated via centrifugation and stored at −80 °C until analysis. Frozen serum was thawed at room temperature and 100 μL serum was mixed with 900 μL 88% methanol containing five internal standards (phenylalanine, caffeine, cholic acid, arachidonic acid, and caffeic acid). The sample was shaken at 2000 rpm for 45 min and then incubated for 2 h in the refrigerator. The mixture was then centrifuged at 14,000 rpm for 10 min at 4 °C. The supernatant solution was transferred, speed-vacuum dried at room temperature, and reconstituted in 20 μL of 50% methanol, ready for injection.
2) **Chromatographic separation and MS conditions:** we used an Agilent 1260 high-performance liquid chromatography system coupled to an Agilent 6540 UHD accurate-mass quadrupole time-of-flight mass spectrometry with dual Jet Stream electrospray ionization (Agilent Technologies, Inc., Santa Clara, CA, US) to separate metabolites in the serum extracts. The injection volume was 2 μL. Analyses were performed in both positive and negative ion modes. Gradient elution with a reverse-phase 50 × 2.1 mm ACQUITY 1.8-μm C18 column (Waters Corporation, Milford, MA, US) in combination with a 2.1 mm × 5 mm, 1.8-μm VanGuard precolumn (Waters Corporation) was performed with mobile phase A (water with 0.1% formic acid) and B (25% isopropanol in acetonitrile with 0.1% formic acid) at 0.5 mL/min, as follows: 0.1%–10% B for 2 min, increased to 99% B for 3 min, hold at 99% B for 2 min, decrease to 0.1% B for 0.3 min, then decrease to 0% B for 0.5 min, hold at 0% B for 1.9 min, and return to the initial condition (0.1% B) for 0.3 min. The column was equilibrated with 0.1% B for 3 min at a flow rate of 0.5 mL/min before the next sample was injected. The temperature of the column and auto-sampler were maintained at 40 °C and 4 °C, respectively. Mass spectrometry was performed in the range of m/z 70–1700 at a scan rate of 4 spectra/second under the following condition: drying gas temperature 300 °C, drying gas flow 8.0 L/min, nebulizer pressure 45 psi, sheath gas temperature 350 °C, sheath gas flow 11 L/min; capillary voltage 4000 V; nozzle voltage 1000 V; fragmenting voltage 100 V; skimmer voltage 45 V; and octopole radio frequency voltage 750 V. Internal mass correction of each sample was performed with continuous infusion of a mixture containing two mass references at 0.05 mL/min through the reference nebulizer. The two reference masses were purine m/z 121.05 and m/z 119.03632; *hexakis* (1*H*,1*H*,3*H*-tetrafluoropropoxy) phosphazine (HP-921) m/z 922.0098 and m/z 966.000725 was used for positive and negative mode, respectively. The full scan data were recorded using Agilent Mass Hunter Data Acquisition software version B.05.00.
3) **MS data analysis and identification:** data processing, including molecular feature extraction, background subtraction, data filtering, and molecular formula estimation were performed using Agilent Mass Hunter Qualitative software version B.06.00. The molecular feature-extracted files were further processed using Agilent Mass Profiler Professional software B.12.6.1 to extract and align peaks/features from the chromatograms of all extracts of the sample. Furthermore, putative compound identification of the features of interest was carried out using a Personal Compound Database Library (Agilent Technologies, Inc.) with the METLIN Personal Metabolite Database.

## 4 Neural network architecture

The construction and training of the artificial neural network were executed on Mathematica version 13.0 (Wolfram, Champaign, IL, USA), a computational software widely used in diverse scientific disciplines due to its advanced capabilities in numerical computation, data visualization, and machine learning tasks. The architecture of the designed neural network was of a feedforward topology, with the input layer comprising of 591 nodes, corresponding to the number of metabolites identified from the metabolic assessments. This design choice aligns with the objective of the study, as each of these nodes represents a distinct metabolite, which will be used to feed the metabolic data into the network for further analysis. Beyond the input layers, the hidden layers of the neural network were designed with a high level of complexity to accommodate the multidimensionality of the data. Specifically, the network contained 10 layers, each hosting 1000 neurons, followed by 35 layers, each accommodating 100 neurons, and finally 19 layers, each consisting of 50 neurons. Each neuron in these layers was designed to process the information from the previous layer, apply a transformation to it, and then pass it on to the next layer. The final layer of the network was a single neuron implementing a linear activation function. This design was adopted because the task at hand – predicting biological age and disease outcomes – is a regression problem, where the output is a continuous value.

### 4.1 Statistical analyses

Baseline characteristics data were shown as mean and standard deviation, mean and standard error or number and percentage as appropriate. Comparisons were performed by the Student’s t test for continuous variables and the Chi squared test for categorical variables. Statistical analysis was performed using predictive models constructed with RStudio version 1.0.136 and R version 3.3.2 (RStudio Inc., Boston, MA, USA). Partial least squares discriminant analysis (PLS-DA) modeling of baseline metabolites for the presence or absence of diabetes at 5 years and mortality over the 11-year study period was performed with the ropls R package for features selection based on the Variable Importance in Projection (VIP) scores.

Analyses of the relationships between independent variables including incident diabetes and mortality with biological age estimated from serum metabolomics, chronological age or serum metabolites were preformed using linear regression analyses. Confounders including BMI, sex, blood pressure smoking status were controlled in the corresponding models where appropriate. We considered P < 0.05 to be statistically significant.

## 5 Results

Table 1A, Table 1B show the clinical characteristics of the study population. The mean age of participants was 40.5 ± 6.4 years, and most were men (70.8%) owing to the demographic structure of the EGAT. All participants had FPG > 97 mg/dL, but none had diabetes at baseline.

**Table 1A:** Clinical characteristics of the study population (n = 454).

Variables	Mean ± standard deviation or n (%)
Age (years)	43.5 ± 6.2
Male sex	393 (86.4%)
Married	436 (96%)
Education at least Bachelor’s	263 (57.9%)
BMI (kg/m^2^)	25.8 ± 3.9
Current or former smoker	203 (50.7%)
Blood pressure >140/90 mmHg	152
Fasting plasma glucose (mg/dL)	104.0 ± 7.4

**Table 1B:** Comparison of clinical characteristics between subjects with and without incident diabetes based on available glycated hemoglobin (n = 404).

Variables	Non-diabetes (n = 343)	Diabetes (n = 61)	P-value
	Mean ± standard error or n (%)	Mean ± standard error or n (%)	
Age (years)	45.5 ± 0.3	44.8 0.9	0.14
Male sex	294 (85.7%)	53 (86.9%)	0.81
Married	330 (96.2%)	59 (96.7%)	0.85
Education at least Bachelor’s	206 (60.1%)	35 (57.4%)	0.69
BMI (kg/m^2^)	25.4 ± 0.2	27.5 ± 0.5	<0.001
Current or former smoker	143 (41.7%)	33 (54.1%)	0.08
Blood pressure >140/90 mmHg	110 (32.1%)	19 (31.1%)	0.89
Fasting plasma glucose (mg/dL)	111.0 ± 1.0	102.7 ± 0.4	<0.001

**Fig. 1:**
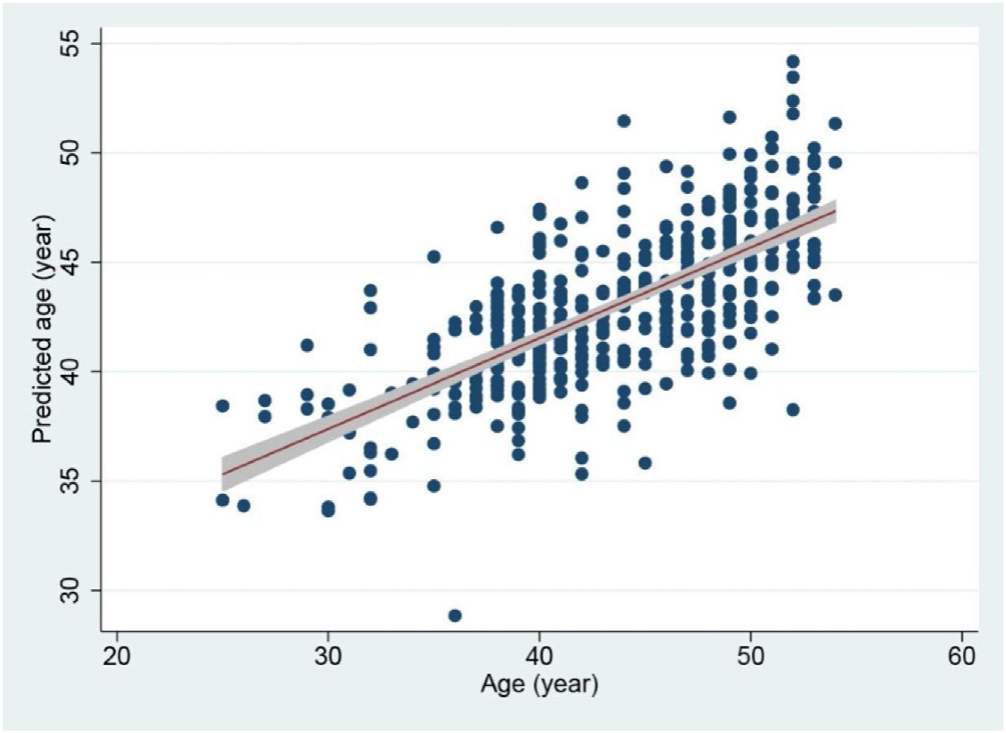
Relationship between chronological age and age predicted using serum metabolites.

**Fig. 2:**
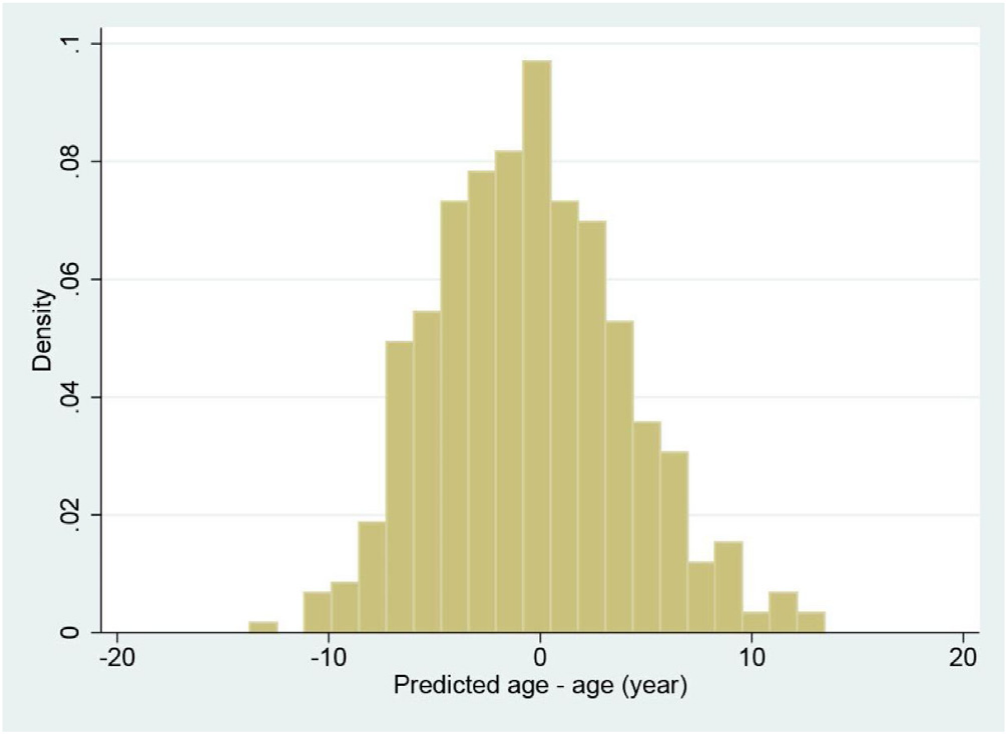
Histogram showing distribution of the difference in predicted age and chronological age.

The relationship between metabolomic age and chronological age is shown in Fig. 1. The line in the figure indicates the linear trend together with the 95% confidence interval (CI). As can be seen in the figure, there was a significant positive correlation between metabolomic age and chronological age (r = 0.71, P < 0.001). The difference between metabolomic age and chronological age is shown as a histogram in Fig. 2. The mean difference was −0.49 years, with a range from −13.7 to +13.4 years.

After 5 years, 404 participants had available glycemic status as assessed using glycated hemoglobin. Sixty-one out of the 404 participants (15.1%) developed diabetes. Table 2A shows the relationship of incident diabetes with chronological age, BMI, and sex. Chronological age tended to be associated with incident diabetes but did not reach statistical significance (P = 0.08) after adjusting for BMI and sex. However, as shown in Table 2B, metabolomic age was significantly related to incident diabetes after controlling for BMI and sex (P < 0.05).

**Table 2A:** Linear regression analysis of the presence or absence of diabetes in 2019 as the independent variable and the chronological age, BMI as well as sex as independent variables.

Variables	OR (95% CI)	P value
Chronological age (years)	1.04 (0.99–1.09)	0.08
BMI (kg/m^2^)	1.15 (1.07–1.23)	<0.001
Female sex	0.96 (0.42–2.0)	0.92

BMI, body mass index; OR, odds ratio; CI, confidence interval.

**Table 2B:** Linear regression analysis of the presence or absence of diabetes in 2019 as the independent variable and the metabolomic age, BMI as well as sex as independent variables.

Variables	OR (95% CI)	P value
Metabolomic age (years)	1.08 (1.00–1.17)	<0.05
BMI (kg/m^2^)	1.14 (1.06–1.22)	<0.001
Female sex	0.92 (0.40–2.09)	0.84

BMI, body mass index; OR, odds ratio; CI, confidence interval.

With regard to mortality, over the 11-year follow-up period, 10 (2.2%) participants died owing to non-accidental causes. As expected, chronological age predicted mortality, independent of BMI and sex (Table 3A). Likewise, metabolomic age was associated with mortality, independent of BMI and sex (Table 3B). However, when metabolomic age and chronological age were included together in the model, metabolomic age, but not chronological age, was independently associated with mortality (Table 3C).

**Table 3A:** Logistic regression analysis of non-accidental mortality during the 9-year follow-up as the independent variable and the chronological age, BMI as well as sex as independent variables.

Variables	OR (95% CI)	P
Chronological age (years)	1.17 (1.05–1.30)	<0.01
BMI (kg/m^2^)	1.03 (0.87–1.23)	0.72
Female sex	0.81 (0.10–6.68)	0.85

BMI, body mass index; OR, odds ratio; CI, confidence interval.

**Table 3B:** Logistic regression analysis of non-accidental mortality during the 9-year follow-up as the independent variable and the metabolomic age, BMI as well as sex as independent variables.

Variables	OR (95% CI)	P
Metabolomic age (years)	1.39 (1.25–1.67)	<0.001
BMI (kg/m^2^)	1.04 (0.89–1.23)	0.62
Female sex	0.71 (0.09–5.90)	0.75

BMI, body mass index; OR, odds ratio; CI, confidence interval.

**Table 3C:** Logistic regression analysis of non-accidental mortality during the 9-year follow-up as the independent variable and the metabolomic age, chronological age, BMI as well as sex as independent variables.

Variables	OR (95% CI)	P
Metabolomic age (years)	1.29 (1.02–1.63)	<0.05
Chronological age (years)	1.07 (0.94–1.22)	0.29
BMI (kg/m^2^)	1.03 (0.87–1.22)	0.71
Female sex	0.76 (0.09–6.35)	0.8

BMI, body mass index; OR, odds ratio; CI, confidence interval.

To further identify baseline metabolites that were related to mortality during the 11-year follow-up, metabolites at baseline were analyzed using PLS-DA and ranked according to variable importance in projection (VIP) scores. Of all identifiable metabolites, we found that beta-D-mannosylphosphodecaprenyl (MPDP) and a number of phosphatidylserines (PSs) were among the five leading metabolites associated with mortality, with VIP scores ranging from 2.26 to 2.88 (Table 4). Using backward stepwise logistic regression, we found that only two PSs and chronological age were independently associated with mortality. The association of beta-D-mannosylphosphodecaprenyl with mortality tended to reach statistical significance (Table 5)

**Table 4:** Five metabolites with top VIP scores.

Metabolites	VIP score
Beta-D-mannosylphosphodecaprenol	2.88
Phosphatidylserine (20:3/0:0)	2.47
Phosphatidylethanolamine (16:0/0:0)	2.35
Phosphatidylserine (20:3/15:0)	2.26
Phosphatidylserine (19:0/0:0)	2.26

**Table 5:** Logistic regression analysis of non-accidental mortality during the 9-year follow-up as the independent variable and the chronological age, sex, BMI, blood pressure, smoking status, phosphatidylserine (20:3/0:0) as well as beta-D-mannosylphosphodecaprenyl as independent variables.

Variables	OR (95% CI)	P
Chronological age (years)	1.20 (1.07–1.35)	<0.01
Female	2.04 (0.17−24.60)	0.57
BMI (kg/m^2^)	1.02 (0.84−1.24)	0.83
Blood pressure >140/90 mmHg	3.29 (0.68−15.68)	0.14
Current or former smoker	1.39 (0.24−8.10)	0.72
Phosphatidylserine (20:3/0:0) (normalized count)	1.76 (1.37–2.25)	<0.001
Beta-D-mannosylphosphodecaprenyl (normalized count)	0.54 (0.28−1.05)	0.1

## 6 Discussion

A number of studies have investigated various predictors of biological age, including telomere length; epigenetic clocks; omics-based predictors such as transcriptomics, proteomics, and metabolomics; and composite biomarker predictors [10]. Of these types of predictors, the epigenetic clock is potentially the current gold standard because it can also predict mortality on top of chronological age [3]. There are relatively few studies exploring metabolomic markers [11,12]. A prior study wherein the metabolic age score, derived from urinary data utilizing 1H NMR spectroscopy, was predictive of survival across an approximate 13-year follow-up peroid [13]. Similarly, van den Akker et al. developed a metabolomics-based age predictor, termed “metaboAge”, using ^1^H NMR blood-based metabolomics to gauge an individual’s biological age. Validations in independent cohorts have shown that when one’s metabolome suggests an age older than their chronological age, there is a heightened associated risk for future cardiovascular diseases, mortality, and compromised functionality in senior individuals [14]. Moreover, in a UK cohort, circulating metabolomes have been used to predict biological age, with performance in predicting diseases that is comparable to that of aging clocks derived from DNA methylation patterns [8]. Our results concur with these previous studies in that we determined that metabolic age, as assessed through serum metabolomics, holds predictive value for the onset of diabetes and mortality outcomes. Taken together, it is likely metabolic age may present a nuanced assessment reflecting the complex interrelation of genetic, environmental, and lifestyle determinants on an individual’s health status. Metabolic age potentially offers an encompassing evaluation of physiological degeneration which could facilitate the identification of individuals at augmented risk, thus enabling more individualized preventive interventions.

In the present study, we found that one of the leading metabolites associated with mortality during the 11-year follow-up was a PS. This association was independent of other known risk factors for mortality such as age, sex, smoking status, and body mass index. The underlying basis for this relationship and its direction are not entirely clear. PS is a phospholipid that is found in high concentrations in the brain, where it plays an important role in neuronal cell death (apoptosis) [15]. Studies have shown that PS is involved in the regulation of apoptosis and that it plays a role in a number of diseases characterized by cell death, such as age-related dementia [16], Parkinson disease [17], and stroke [18]. PS supplements have been shown to improve cognition and memory in healthy adults, as well as in those with Alzheimer disease and other forms of dementia [19]. Additionally, PS containing liposomes have been shown to reduce brain inflammation in an animal model of surgical brain injury [20]. Given the aforementioned results, it is likely that the positive association between PS and mortality may not be causal in nature. For example, it is possible that PS is a marker of underlying disease risk rather than a cause of mortality. Additionally, reverse causality may be present, meaning that individuals who are nearing the end of their life may have higher levels of circulating phosphatidylserine owing to the increased apoptosis that occurs during this time. Future studies that use a prospective design are warranted to more accurately assess the relationship between PS and mortality.

In the present study, we found that serum MPDP was related to future mortality; however, the underlying basis of this relationship is unclear. MPDP is a metabolite in the lipoarabinomannan (LAM), a cell wall component of *Mycobacterium tuberculosis* biosynthesis [21]. LAM has a role in modulation of the immune response via involvement in the inhibition of phagosome maturation, apoptosis, and interferon-γ signaling in macrophages and interleukin-12 cytokine secretion in dendritic cells [22]. Recent progress in the identification of genes involved in LAM biosynthesis has illustrated that enzymes controlling the LAM/LM (lipoarabinomannan/lipomannan) balance might represent targets for new antitubercular drugs [23]. It is conceivable that alteration of the enzymatic pathways in LAM biosynthesis might result in higher MPDP levels and decreased LAM, hence rendering the host less susceptible to mycobacterium infection and death, particularly owing to tuberculosis, which is prevalent in a number of countries and regions, including Thailand.

The present study has a number of limitations that should be considered. First, the study population was relatively healthy and did not have diabetes at baseline. Our results may differ in a population with more chronic diseases. Moreover, other potentially relevant variables such as chronic inflammation or other chronic diseases were not available for the analyses. Additionally, this study relied on a single measurement of metabolites at baseline, and metabolite levels may fluctuate over time. Future studies should use repeated measures of metabolites to more accurately assess their role in mortality risk. Additionally, the present study was not designed to assess the causal relationship between metabolites and mortality. Prospective studies are needed to accurately assess this relationship. Despite these limitations, the present study has a number of strengths. The study included a reasonably large population, and the follow-up period was relatively long. Additionally, we used a novel method to assess biological age and its relationship with mortality. This method allowed for assessment of the relationship between metabolites and mortality.

## Authors’ contributions

Conceptualization, Boonsong Ongphiphadhanakul; Formal analysis, La-or Chailurkit and Boonsong Ongphiphadhanakul; Funding acquisition, Prin Vathesatogkit; Investigation, La-or Chailurkit, Nisakron Thonmung, Prin Vathesatogkit, Piyamitr Sritara and Boonsong Ongphiphadhanakul; Methodology, La-or Chailurkit, Nisakron Thonmung and Prin Vathesatogkit; Supervision, Piyamitr Sritara and Boonsong Ongphiphadhanakul; Validation, Boonsong Ongphiphadhanakul; Writing – original draft, La-or Chailurkit, Prin Vathesatogkit, Piyamitr Sritara and Boonsong Ongphiphadhanakul.

## Ethical standards

The Committee on Human Rights Related to Research Involving Human Subjects, Faculty of Medicine, Ramathibodi Hospital, Mahidol University approved this study (No. 2558/729). The study conformed to the provisions of the Declaration of Helsinki (as revised in Fortaleza, Brazil, October 2013). All participants gave their written informed consent before participating in the study.

## Conflicts of interest

The authors declare no conflicts of interest regarding the publication of this paper.
